# Indole-3-acetic acid derived from *Blautia* protects against sepsis-induced acute lung injury

**DOI:** 10.3389/fimmu.2026.1707493

**Published:** 2026-03-23

**Authors:** Ke Chao, Yuchao Ding, Wenxuan Ji, Dong Wang, Qi Liang, Shilong Sun, Lifeng Li, Hongfu Yang, Jie Zhao

**Affiliations:** 1 National Engineering Laboratory for Internet Medical Systems and Applications, The First Affiliated Hospital of Zhengzhou University, Zhengzhou, Henan, China; 2 Department of General Medicine, The First Affiliated Hospital of Zhengzhou University, Zhengzhou, Henan, China; 3 Intensive Care Unit, The First Affiliated Hospital of Zhengzhou University, Zhengzhou, Henan, China

**Keywords:** *Blautia*, gut microbiota, indole-3-acetic acid, sepsis, sepsis-associated acute lung injury

## Abstract

**Objectives:**

Sepsis-induced acute lung injury (SI-ALI) significantly contributes to sepsis mortality, with CD8^+^ T cell depletion being a critical pathogenic factor. While *Blautia*, a gut commensal bacterium with established probiotic benefits in diverse diseases, its role in SI-ALI pathogenesis remains undefined. Here, we investigated the therapeutic potential of *Blautia* in lethal SI-ALI.

**Methods:**

Gut microbiome profiling was performed in SI-ALI patients and healthy controls to identify disease-associated microbial alterations. A cecal ligation and puncture (CLP) rat model of sepsis was used to validate microbiota changes and evaluate the therapeutic effects of *Blautia* supplementation. Untargeted metabolomic analysis was conducted to identify key metabolites associated with *Blautia*. Functional studies were performed to assess lung injury, immune responses, CD8^+^ T cell abundance, and survival following *Blautia* or metabolite administration.

**Results:**

Gut microbiome analysis identified significant *Blautia* depletion in SI-ALI patients compared to healthy controls. This pathogenic alteration was faithfully reproduced in cecal ligation and puncture (CLP) -modeled septic rats, in which *Blautia* supplementation attenuated lung injury, enhanced systemic immune responses,and improved survival. Untargeted metabolomic profiling identified indole-3-acetic acid (IAA) as a key *Blautia*-derived metabolite. Administration of IAA alone recapitulated the protective effects of *Blautia*, significantly ameliorating SI-ALI. Crucially, both interventions restored CD8^+^ T cell populations and augmented their functional responses. Clinical analysis revealed that elevated *Blautia* abundance in sepsis patients inversely correlated with pulmonary injury severity and positively associated with augmented CD8^+^ T cell effector functions.

**Discussion:**

Our findings establish that *Blautia* and *Blautia*-derived IAA mitigates SI-ALI by counteracting CD8^+^ T cell depletion and dysfunction, highlighting a novel and promising mechanism-based therapeutic strategy for life-threatening sepsis.

## 1 Introduction

Sepsis, characterized by a dysregulated host response to infection that precipitates life-threatening multiorgan failure, remains one of the most critical medical challenges worldwide (1–3). The sepsis cytokine storm triggers an excessive inflammatory storm, often leading to acute lung injury(ALI) (4). During sepsis, dysregulated immune responses are the most prevalent pathological phenomenon, characterized by cytokine storm, acquired immunosuppression, metabolic disorders, and multi-organ failure (5–9). Despite therapeutic targeting of systemic inflammation, outcomes in SI-ALI remain suboptimal, largely due to the complexity and heterogeneity of individual immune responses (10). Therefore, new immunomodulatory therapies are urgently needed to improve the prognosis of patients with SI-ALI.

Recent studies establish the gut microbiota as a key regulator of sepsis pathophysiology. Critically, it drives multi-organ dysfunction via complex inter-organ crosstalk, primarily mediated by translocation of live bacteria, metabolites, and various mediators, such as pathogen-associated molecular patterns (PAMPs), and damage-associated molecular patterns (DAMPs) into systemic circulation. Growing evidence indicates that strategic modulation of gut microbiota composition and metabolites offers therapeutic potential for sepsis management (11–13).

*Blautia*, predominantly found in mammalian feces and the intestinal tract, is a gram-positive anaerobic bacterium implicated in tumor progression and remodeling of the immune microenvironment (14). *Blautia* abundance is negatively correlated with a variety of diseases, including autoimmune diseases and metabolic syndrome (15–17). For example, *Blautia* can induce adaptive intestinal immune responses during homeostasis and produce bioactive molecules that can modulate long-range functional cellular activities and participate in pathological development (18–20). Furthermore, *Blautia* has been shown to ameliorate metabolic disorders such as obesity and diabetes in humans and mice (21). Therapeutic administration of *Blautia* and its metabolite acetate enhances CD8^+^ T cell infiltration and function, amplifying antitumor immunity in breast and colorectal cancer (22, 23). However, its effects on SI-ALI and the underlying mechanisms remain unknown. Clinical and experimental studies have reported associations between altered Blautia abundance and disease severity or outcomes in infectious conditions, including bacterial infections and sepsis, suggesting a potential role in modulating host responses to infection (24, 25). Accumulating evidence has demonstrated that sepsis is accompanied by profound alterations in gut microbiota composition, characterized by reduced microbial diversity, depletion of obligate anaerobes, and expansion of opportunistic pathogens. These microbiota changes have been associated with disease severity, immune dysregulation, and clinical outcomes in both experimental models and patient cohorts. Notably, Blautia has repeatedly emerged as a sepsis-associated taxon, and recent studies have identified alterations in Blautia abundance as a potential microbial indicator of sepsis and sepsis-related outcomes (26). However, despite its recurrent association with sepsis, the functional significance of Blautia and its related metabolic features during sepsis-induced acute lung injury remains incompletely understood. However, current evidence is largely correlative, and the mechanistic contribution of Blautia to infection-associated immune dysfunction remains to be fully elucidated.

This study reveals a novel mechanism explaining the immunomodulatory effects of *Blautia* in a preclinical sepsis model. Our findings demonstrate altered gut *Blautia* abundance in patients with SI-ALI, implicating this genus in disease pathogenesis. *In vivo* studies demonstrate that *Blautia*-derived IAA rescues sepsis-induced CD8^+^T cell dysfunction and confers protection against lethal SI-ALI.

## 2 Materials and methods

### 2.1 Fecal and blood sample collection

Fecal and serum samples from patients with SI-ALI were collected at the First Affiliated Hospital of Zhengzhou University between 2021 and 2023.The control group samples were from demographically matched healthy adults. Sepsis was diagnosed according to the Sepsis-3 definitions, defined as life-threatening organ dysfunction caused by a dysregulated host response to infection, with organ dysfunction identified by an increase in the Sequential Organ Failure Assessment (SOFA) score of ≥2 points from baseline. Sepsis-induced acute lung injury (SI-ALI) was diagnosed based on the Berlin criteria for acute respiratory distress syndrome (ARDS), including acute onset, bilateral pulmonary infiltrates on chest imaging not fully explained by cardiac failure or fluid overload, and impaired oxygenation. Patients with pre-existing chronic lung diseases (including chronic obstructive pulmonary disease or pulmonary fibrosis), active malignancy, autoimmune disorders, long-term immunosuppressive therapy, or incomplete clinical data were excluded from the study. The initial human gut microbiome analysis was conducted in a small pilot cohort to identify candidate microbial signatures for hypothesis testing in subsequent control models.

The majority of enrolled SI-ALI patients had received broad-spectrum antibiotics prior to sample collection. Treatment duration ranged from 3 to 7 days prior to fecal sample collection. Most patients were on enteral nutrition support; a subset with severe gastrointestinal dysfunction received partial or total parenteral nutrition. Peripheral blood samples were collected in strict compliance with medical ethics protocols. Venous blood was drawn from SI-ALI patients for the isolation of peripheral blood mononuclear cells (PBMCs) for subsequent flow cytometry analysis. All samples were processed within 30 minutes of collection to ensure integrity prior to cryopreservation at -80°C.

### 2.2 Bacterial culture and preparation

*Blautia* coccoides strain BNCC 361193 (Guangzhou Zuoke Biotechnology, China) was anaerobically cultured in sterile Reinforced Clostridium Medium (ATCC 2722) supplemented with tryptic soy broth, yeast extract, L-cysteine·HCl, hemin, and vitamin K1 at 37 °C for 4–5 days. Bacterial cultures were harvested during the exponential growth phase at an OD600 of approximately 1.0 for downstream applications.

### 2.3 Animal experiments

Adult male Sprague-Dawley rats (approximately 200 g) were provided by Beijing Weitong Lihua Laboratory Animal Technology Co., Ltd. After one week of acclimation, the rats were randomly divided into three groups: sham operation (Sham group), CLP-induced sepsis-related ALI group (CLP group), and *Blautia*/IAA (MCE, USA)-treated group (CLP+*Blautia*/CLP+IAA group). This model was established based on previous reports (27). All rats were resuscitated with subcutaneous injection of normal saline (37 °C) and warm light. Survival was observed within 72 hours after CLP, and lung tissue and feces were collected 72 hours after surgery. All rats were antibiotic-naïve and had free access to food and water prior to and following surgical procedures. Thereafter, the rats were housed in a constant-temperature animal room with a 12-h light/12-h dark cycle. Feces were stored in a liquid nitrogen tank until further use. Lung tissue was fixed overnight with 4% H2 paraformaldehyde for pathological examination.

### 2.4 Histopathological analysis of lung

The paraformaldehyde-fixed lung tissues were embedded in paraffin and cut into 5-µm sections. The lung sections were stained with hematoxylin and eosin using the standard protocol.

### 2.5 Lung wet-to-dry weight ratio

The wet-to-dry weight ratio of lung tissue serves as an indicator for assessing pulmonary edema formation. To determine this ratio, the right middle lobe was harvested and immediately weighed to obtain the wet weight. The tissue was then placed in an oven at 60 °C for 48 hours. After complete desiccation, the lung tissue was re-weighed to record the dry weight. The wet-to-dry weight ratio was calculated to evaluate the extent of pulmonary tissue edema.

### 2.6 High-throughput sequencing of the 16S ribosomal RNA gene

The V3-V4 hypervariable regions of 16S rRNA were amplified using universal primers 338F (5’-ACTCCTACGGGAGGCAGCAG-3’) and 806R (5’-GGACTACHVGGGTWTCTAAT-3’) through polymerase chain reaction (PCR). Subsequent high-throughput sequencing was performed on the Illumina NovaSeq 2000 platform (PE300 mode) by Shanghai Majorbio Bio-pharm Technology Co., Ltd. To ensure data quality, rigorous quality control measures were implemented throughout all experimental procedures, including template DNA extraction, PCR amplification, product purification, library construction, and sequencing processes. This comprehensive quality assurance approach guaranteed the generation of highly accurate and reliable sequencing data for downstream microbial community analysis. Briefly, differential taxa identified by LEfSe analysis (LDA score > 2.0, P < 0.05) were classified as either enriched or depleted in the disease group relative to controls. The MDI for each sample was defined as the log-transformed ratio of the cumulative relative abundance of taxa enriched in the disease group to that of taxa depleted in the disease group. Higher MDI values indicate a greater extent of microbial dysbiosis. Group differences in MDI were assessed using the Wilcoxon rank-sum test, and correlations between MDI and microbial community structure were interpreted in conjunction with Bray–Curtis-based PCoA and PERMANOVA analyses. MDI=log10[(total abundance in genera increased in disease group)/(total abundance in genera decreased in disease group)].

### 2.7 Metabonomic data acquisition

The collected fecal samples (25 mg per rat) were ground and added into a centrifuge tube. Fecal extract (500 µL) was added into the tube and the sample was mixed well by vortex and incubated at -40°C for 1 h. Following incubation, the sample was centrifuged at 4°C and 12000 rpm for 15 min, and the supernatant was collected for metabolomics analyses. Mass spectrometry (MS) analysis was performed using the HPLC-MS/MS platform with the positive and negative ion modes (POS and NEG, respectively).

### 2.8 Multivariate data analysis

Following LC-MS analysis, raw data were processed using Progenesis QI (Waters Corporation, Milford, USA) to generate a data matrix of retention times, mass-to-charge ratios (m/z), and peak intensities. Metabolite identification was achieved by matching MS and MS/MS spectra against public databases HMDB (http://www.hmdb.ca/), Metlin (https://metlin.scripps.edu/), and Majorbio’s self-built database. The data matrix underwent preprocessing involving removal of missing values, imputation of null entries using the observed minimum value, and sum normalization applied to peak response intensities to mitigate technical variations arising from sample preparation and instrument instability. Variables exhibiting >30% relative standard deviation (RSD) in quality control (QC) samples were subsequently filtered out. Significantly differential metabolites were identified based on combined criteria of variable importance in projection (VIP)>1 from orthogonal partial least squares-discriminant analysis (OPLS-DA) and statistical significance (p<0.05) determined by Student’s t-test. Differential metabolites were visualized through volcano plots and heatmaps, followed by metabolic pathway annotation using the KEGG database (https://www.kegg.jp/kegg/pathway.html) to elucidate their associated biological pathways.

### 2.9 Real-time quantitative PCR

To detect the abundance of *Blautia* in the intestinal microbiota of SI-ALI patients, fecal DNA was extracted from patient stool samples using the TIANamp Stool DNA Kit (DP328, TIANGEN, China) according to the manufacturer’s instructions. Real-time quantitative PCR (RT-qPCR) was performed using the SYBR Green Premix Pro Taq HS qPCR Kit (AG11718, Accurate Biology, China) on a QuantStudio™ 5 Real-Time PCR System (Applied Biosystems, USA). The 16S rRNA gene was used as the internal reference. The primer sequences were as follows:*Blautia*: F-CGGTACCTGACTAAGAAG; R-AGTTT(C/T)ATTCTTGCGAAC. 16s:F-CGGCAACGAGCGCAACCC; R-CCATTGTAGCACGTGTGTAGCC.

### 2.10 Flow cytometry protocol

Cells were analyzed ex vivo without additional stimulation, and intracellular IFN-γ expression reflects *in vivo* T-cell activation during sepsis. Cells isolated from peripheral blood and spleen were incubated with fluorochrome-conjugated surface antibodies (CD3, CD4, CD8) (Biolegend, USA) for 30 minutes in the dark. Subsequently, cells were fixed with 4% paraformaldehyde (PFA) buffer (Servicebio, China) for 20–30 minutes, followed by permeabilization using a membrane permeabilization agent (Biolegend, USA) for 30 minutes. Finally, intracellular staining was performed using antibodies against Granzyme B and IFN-γ (Biolegend, USA) for 30 minutes. Flow cytometry analysis was performed using a FACSCanto II flow cytometer (BD, USA).

### 2.11 Statistical analysis

Statistical analysis was performed using GraphPad Prism 9.5 (GraphPad Software, USA). Data in this study were expressed as mean ± SEM. The experimental data were calculated by the two-tailed unpaired Student’s t- test or one- way analysis of variance (ANOVA). The survival rate differences of animals were analyzed by log- rank (Mantel- Cox) test. The critical p value was set to 0.05 for significant differences.

## 3 Results

### 3.1 Intestinal *Blautia* participates in SI-ALI progression in humans

**Figure 1:**
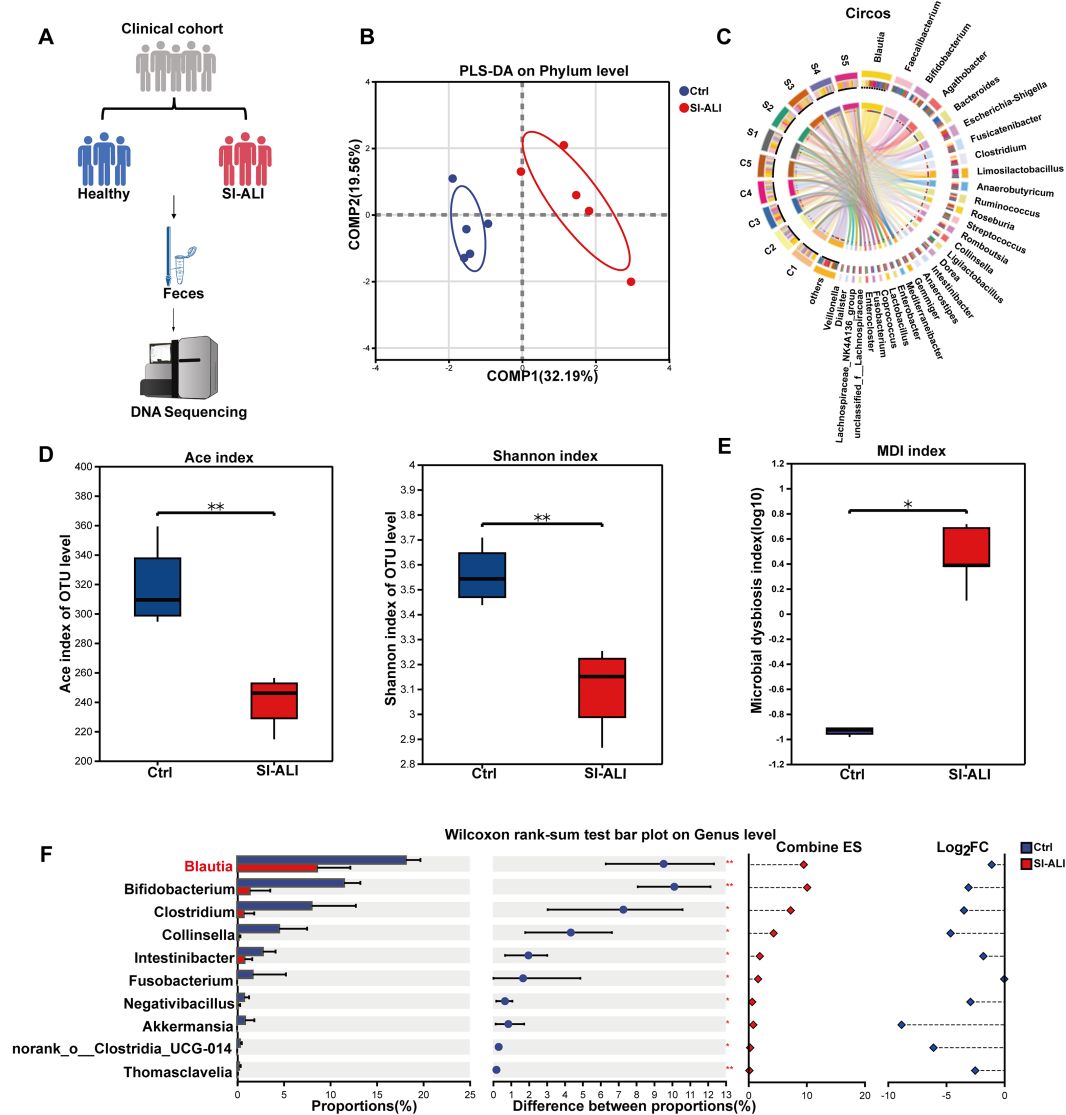
Intestinal *Blautia* participates in SI-ALI progression in humans. **(A)** Flowchart of patient recruitment, group allocation, and analytical procedures. **(B)** Partial Least Squares Discriminant Analysis (PLS-DA) scatter plot of fecal samples from healthy controls and SI-ALI patients (phylum level). **(C)** Circos plot delineating taxon-sample relationships in SI-ALI and healthy controls. (C=Ctrl, S=SI-ALI) **(D)** Alpha Diversity of gut microbiota in healthy controls and SI-ALI patients (Ace index and Shannon index). **(E)** Comparative analysis of the Microbial Dysbiosis Index (MDI) between healthy controls and SI-ALI patients. **(F)** Differential gut microbiota at the genus level between SI-ALI versus healthy controls. * P<0.05; ** P<0.01.

To investigate the interaction between gut microbiota and SI-ALI, this study conducted an exploratory analysis of genomic DNA from fecal samples of SI-ALI patients (n=5) and healthy controls (n=5) in a small pilot clinical cohort, and performed high-throughput sequencing (**Figure 1A**, **Supplementary Table 1**). Using partial least squares discriminant analysis (PLS-DA), we demonstrated significant differences in gut microbiota composition at the phylum level between SI-ALI patients and healthy controls (**Figure 1B**). Circos visualization software was used to plot taxon-sample association maps in SI-ALI and healthy controls to analyze microbial community structure. Predominant taxa (relative abundance ≥0.01) exhibited cohort-specific clustering, while low-abundance taxa (<0.01) were classified as “other.” A total of 652 distinct taxonomic entities were identified across all samples (**Figure 1C**). SI-ALI patients exhibited significantly lower gut microbiota diversity and community richness than controls (**Figure 1D**). Furthermore, the microbial dysbiosis index (MDI) revealed significantly higher levels of gut microbiota dysbiosis in SI-ALI patients compared with controls (**Figure 1E**). At the genus level, the relative abundance of bacterial communities in SI-ALI patients was reduced compared with healthy controls, such as *Blautia*, *Bifidobacterium*, and *Akkermansia*, with the decrease in *Blautia* being particularly significant (**Figure 1F**). Taken together, these findings suggest that SI-ALI patients exhibit severe intestinal dysbiosis. While the significant reduction in Blautia levels may be influenced by clinical confounders common in critical illness-most notably broad-spectrum antibiotic therapy-it is implicated as a potential contributor to the pathophysiological progression of SI-ALI. To isolate the specific effect of sepsis pathophysiology, we subsequently employed an antibiotic-naïve animal model.

### 3.2 Intestinal *Blautia* is involved in the progression of SI-ALI in rats

**Figure 2:**
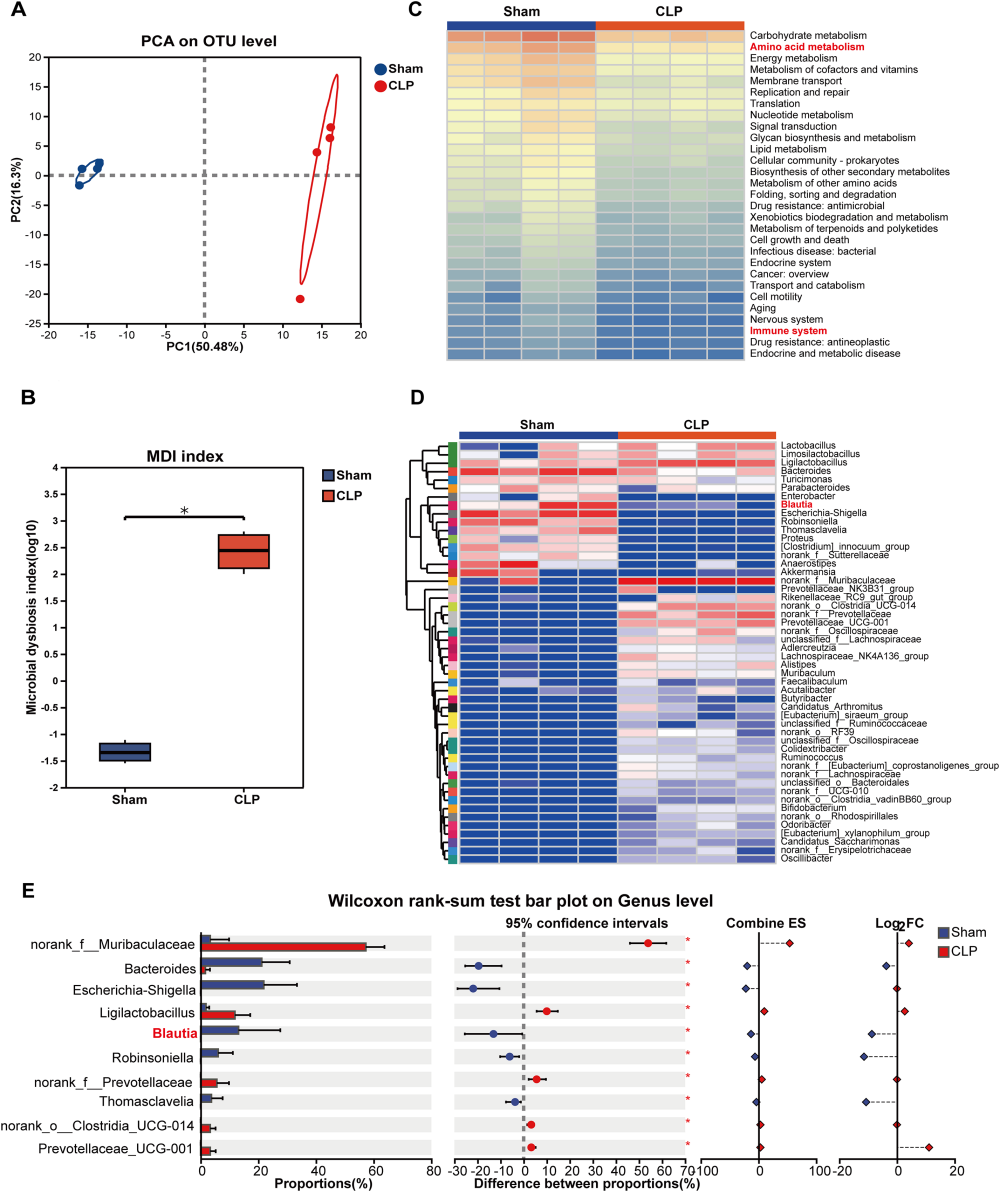
Intestinal *Blautia* is involved in the progression of SI-ALI in rats. **(A)** Principal component analysis (PCA) of gut microbiome in Sham and CLP rats at the OTU level (fecal samples were collected 72 hours after CLP surgery). **(B)** Comparative analysis of MDI between sham controls and the CLP group. **(C)** Functional analysis of intestinal flora in CLP-induced ALI rats and sham-operated group based on PICRUSt2. **(D)** Relative abundance of gut microbial species in sham controls and CLP Models (genus level). **(E)** Differences in intestinal microbiota composition at the genus level between sham-operated and CLP-induced SI-ALI rats. * P<0.05.

To further characterize the dynamics of *Blautia* during SI-ALI progression, we established a rat SI-ALI model. Principal component analysis (PCA) of fecal samples from sham-operated and CLP-induced SI-ALI rats revealed significant differences in gut microbiota composition between the two groups (**Figure 2A**). Consistent with observations in human sepsis, MDI analysis revealed significantly higher MDI scores in the CLP-induced SI-ALI group than in the sham-operated group, suggesting that gut microbiota dysbiosis may be a direct pathophysiological consequence of SI-ALI progression (**Figure 2B**). Furthermore, PICRUSt2 functional prediction revealed enrichment of metabolic pathways during SI-ALI pathogenesis. Gut microbiota analysis revealed functional and compositional perturbations in SI-ALI rats, with 32 metabolic pathways demonstrating significant enrichment (**Figure 2C**). Amino acid metabolism and immune-related pathways exhibited the most pronounced changes. (**Figure 2C**). Next, we sought to confirm the specific depletion of *Blautia* in the rat SI-ALI model. This analysis confirmed a significant decrease in the abundance of *Blautia* in the CLP group, as clearly visible in the genus-level heatmap (**Figure 2D**). Interestingly, we found that the expression of some beneficial bacteria (such as Lactobacillus) was upregulated, while the expression of some potentially harmful bacteria (such as Escherichia coli/Shigella) was decreased. This change may reflect a transient dynamic response of the gut ecosystem to acute sepsis stress, rather than a stable restoration of microbial homeostasis. Although Lactobacillus is generally considered a beneficial commensal, its enrichment under sepsis conditions may reflect its ecological adaptation to inflammatory stress, rather than its protective function. The decreased abundance of Escherichia coli-Shigella in the gut does not rule out its translocation or systemic pathogenicity during sepsis. Genus-level differential abundance analysis revealed that *Blautia*, *Escherichia coli-Shigella*, and *Bacteroides* were all significantly reduced in the CLP group (**Figure 2E**). These findings demonstrate that enteric *Blautia* are players in the pathophysiology of sepsis, with their relative abundance dynamically modulated during sepsis progression.

### 3.3 *Blautia* reduces SI-ALI by enhancing CD8^+^ T cell function

**Figure 3:**
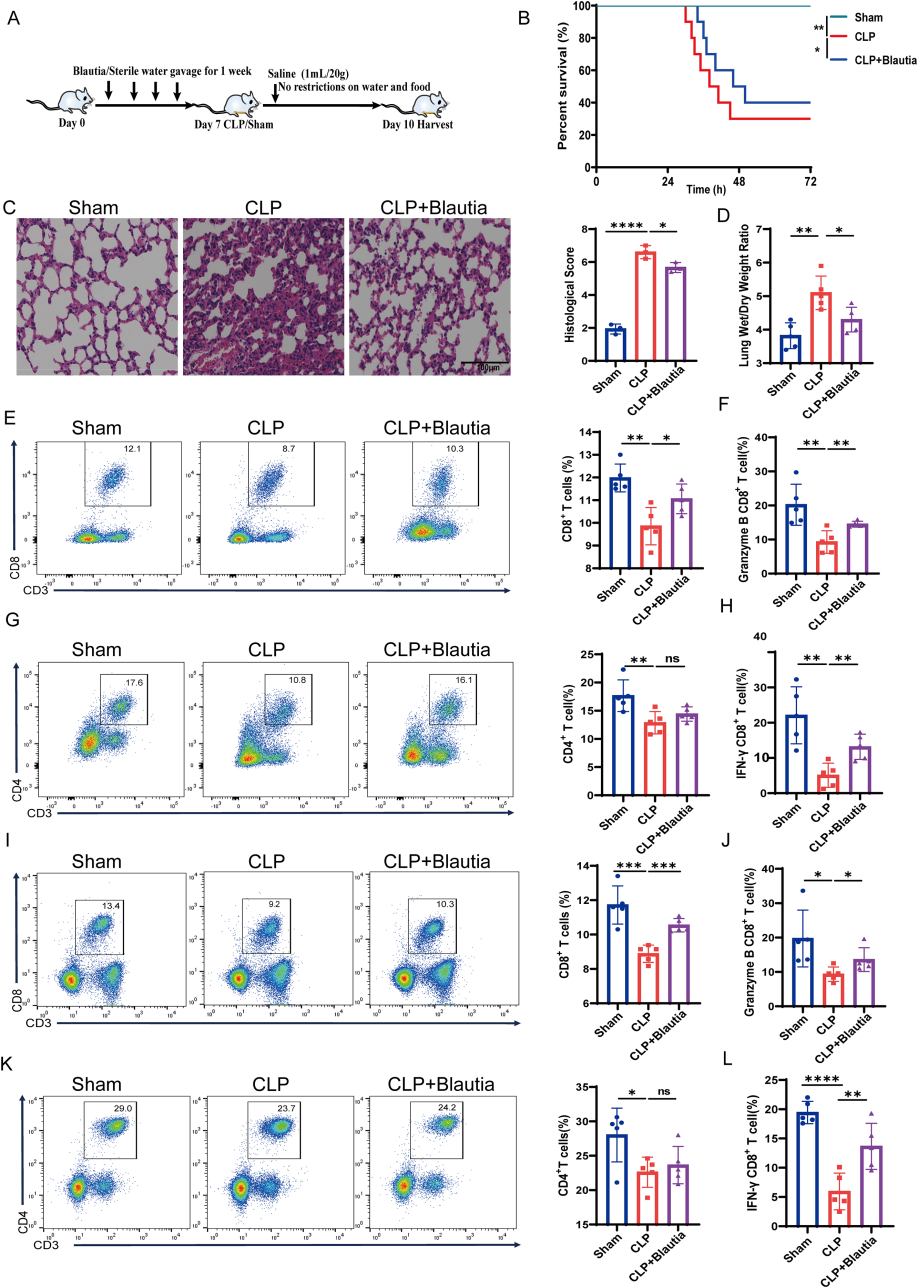
*Blautia*-Mediated Attenuation of SI-ALI via Enhanced CD8^+^ T Cell Founction. **(A)** Experimental grouping and treatment flowchart for rats. **(B)** Kaplan-Meier survival analysis: sham-operated group, CLP-induced SI-ALI group, and *Blautia*-pretreated group. **(C)** Representative H&E-stained rat lung sections and histological scores of Sham-Operated, CLP, and *Blautia*-Pretreated Cohorts. **(D)** Comparison of the Lung Wet/Dry Weight Ratio of rats in each group. **(E-H)** Peripheral blood T cell frequency and cytokine production: CD3^+^CD8^+^ populations, CD3^+^CD4^+^ populations, IFN-γ^+^CD8^+^ subsets, and Granzyme B^+^CD8^+^ subsets in sham-operated group, CLP rats, and *Blautia*-Pretreated CLP Groups. **(I-L)** Splenic T cell frequency and effector function: CD3^+^CD8^+^ populations, CD3^+^CD4^+^ populations, IFN-γ^+^CD8^+^ subsets, and Granzyme B^+^CD8^+^ subsets in sham-operated group, CLP rats, and *Blautia*-Pretreated CLP Groups. * P<0.05; ** P<0.01; *** p<0.001; **** p<0.0001.

Next, to determine the direct effects of *Blautia* on SI-ALI, we treated rats with *Blautia* or sterile water and then underwent sham or CLP surgery (**Figure 3A**). Rats pretreated with *Blautia* had slightly improved survival compared with rats challenged with CLP (**Figure 3B**). Concurrently, we evaluated the effects of *Blautia* on SI-ALI. Compared with the sham group, rats in the CLP group exhibited severe lung inflammation, alveolar wall thickening, and inflammatory cell infiltration. Histological analysis revealed that lung injury was attenuated in *Blautia*-treated rats compared with the CLP group (**Figure 3C**). The lung wet/dry weight ratio, a measure of lung injury severity, was highest in the CLP group, while pretreatment with *Blautia* attenuated lung injury in rats (**Figure 3D**). Recent studies have demonstrated a significant correlation between SI-ALI and immunosuppression (28, 29). **Figure 2C** shows a strong correlation between altered gut microbiota composition and impaired immune function during SI-ALI. In summary, our results suggest that *Blautia* may attenuate lung injury by modulating immune responses. Flow cytometric analysis of blood and spleen samples revealed a significant decrease in both CD8^+^ T cell and CD4^+^ T cell populations in the CLP group compared with the sham group, indicating immunosuppression in SI-ALI rats. *Blautia* pretreatment restored CD8^+^ T cell infiltration to near-normal levels, whereas no statistically significant change was observed in the proportion of CD4^+^ T cells compared to the CLP group. These findings suggest that *Blautia* improves SI-ALI-induced immunosuppression primarily by regulating CD8^+^ T cells (**Figures 3E, G, I, K**). Furthermore, functional flow cytometric analysis revealed impaired CD8^+^ T cell function in the peripheral blood and spleen of the CLP group compared with the sham group. *Blautia* pretreatment increased frequency of IFN-γ CD8^+^ T cells and granzyme B CD8^+^ T cells in SI-ALI rats, while alleviating immunosuppression (**Figures 3E–L**). Together, these findings suggest that *Blautia* ameliorates SI-ALI by enhancing the number and function of host CD8^+^ T cells.

### 3.4 *Blautia*-derived IAA is involved in the pathophysiological process of SI-ALI development

**Figure 4:**
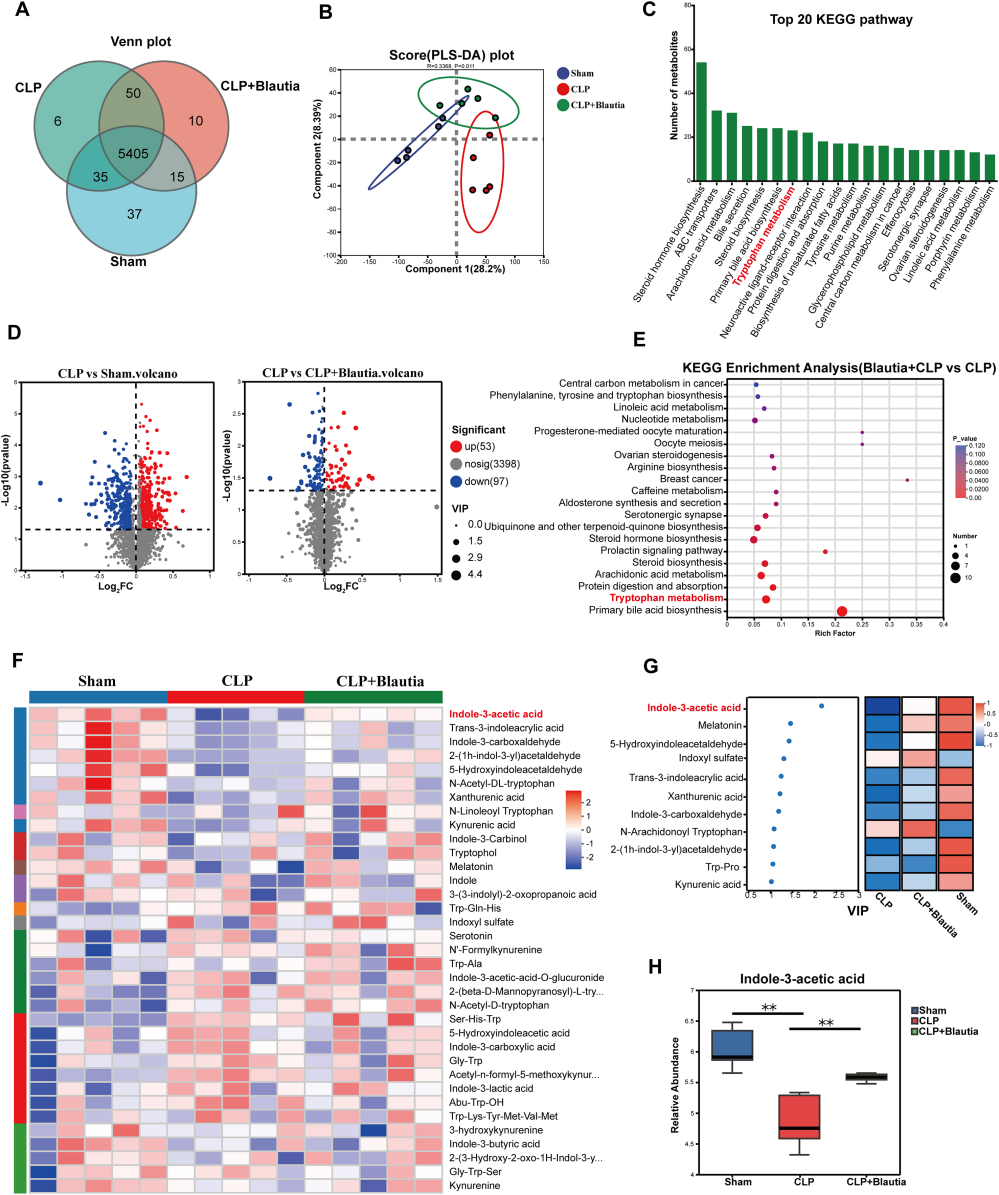
*Blautia*-derived IAA is involved in the pathophysiological process of SI-ALI development. **(A)** Venn diagram of metabolites shared and unique among Sham, CLP, and *Blautia*-Pretreated CLP rats. **(B)** PLS-DA scatter plot of fecal metabolomes: Sham, CLP, and *Blautia*-Pretreated CLP rats. **(C)** Top 20 differentially enriched metabolic pathways in gut microbiota gene sets among cohorts. **(D)** Differential fecal metabolite analysis: Sham *vs*. CLP-Septic Rats and CLP-*vs*. *Blautia*-Intervention Cohorts (Volcano plot representation). **(E)** KEGG enrichment analysis of intestinal metabolites: CLP group versus *Blautia*-Pretreated CLP group. **(F)** Heatmap visualization of differential metabolites: Sham-operated, CLP group, and *Blautia*-Pretreated CLP groups (color key: blue = downregulation; red = upregulation). **(G)** Discriminant metabolites identified by VIP scoring. **(H)** Quantification metabolomics of IAA levels. ** P<0.01.

To elucidate the mechanism by which reduced *Blautia* abundance alleviates SI-ALI, metabolomic analysis of fecal metabolome extracts from the CLP group was performed. Preliminary metabolomic analysis identified 5405 common metabolites in the sham, CLP, and CLP + *Blautia* groups (**Figure 4A**). Subsequent PLS-DA analysis and volcano plots revealed distinct metabolite profiles among the three experimental groups (**Figures 4B, D**). The metabolites identified in this study were significantly enriched in key KEGG pathways, including steroid and bile acid metabolism (steroid biosynthesis, steroid hormone biosynthesis, primary bile acid biosynthesis, and bile secretion), lipid signaling and transport (arachidonic acid metabolism, glycerophospholipid metabolism, and ABC transporters), neuroactive ligand-receptor interactions, and tryptophan metabolism (**Figure 4C**). Comparative metabolomic analysis revealed a significant enrichment of the tryptophan metabolism pathway in the *Blautia* pretreatment group compared with the CLP group (**Figure 4E**). Our data demonstrated that Blautia pretreatment primarily altered tryptophan metabolic pathways in SI-ALI rats. Among the significantly upregulated tryptophan-related metabolites (including indole, indole-3-acetamide, and tryptamine), the increase in indole-3-acetic acid (IAA) was quantitatively the most pronounced and statistically the most significant (VIP score > 2.0, **p < 0.01; see **Supplementary Table 2** for full list) (**Figures 4F, G**). IAA is considered a common plant auxin and microbiota-derived indole metabolite produced by the gut microbiota and has demonstrated potential therapeutic benefits for various lung diseases (30). To further investigate changes in IAA during SI-ALI progression, LC-MS-based metabolomics with relative quantification revealed that IAA levels were significantly reduced in the CLP group compared with the sham group. *Blautia* pretreatment restored IAA biosynthesis to near-physiological concentrations in CLP rats (**Figure 4H**). Collectively, these data indicate that increased Blautia abundance is associated with elevated intestinal IAA levels during sepsis, suggesting a potential link between Blautia-related microbiota changes and IAA-associated host responses.

### 3.5 *Blautia*-derived IAA reduces SI-ALI by enhancing CD8^+^ T cell function

**Figure 5:**
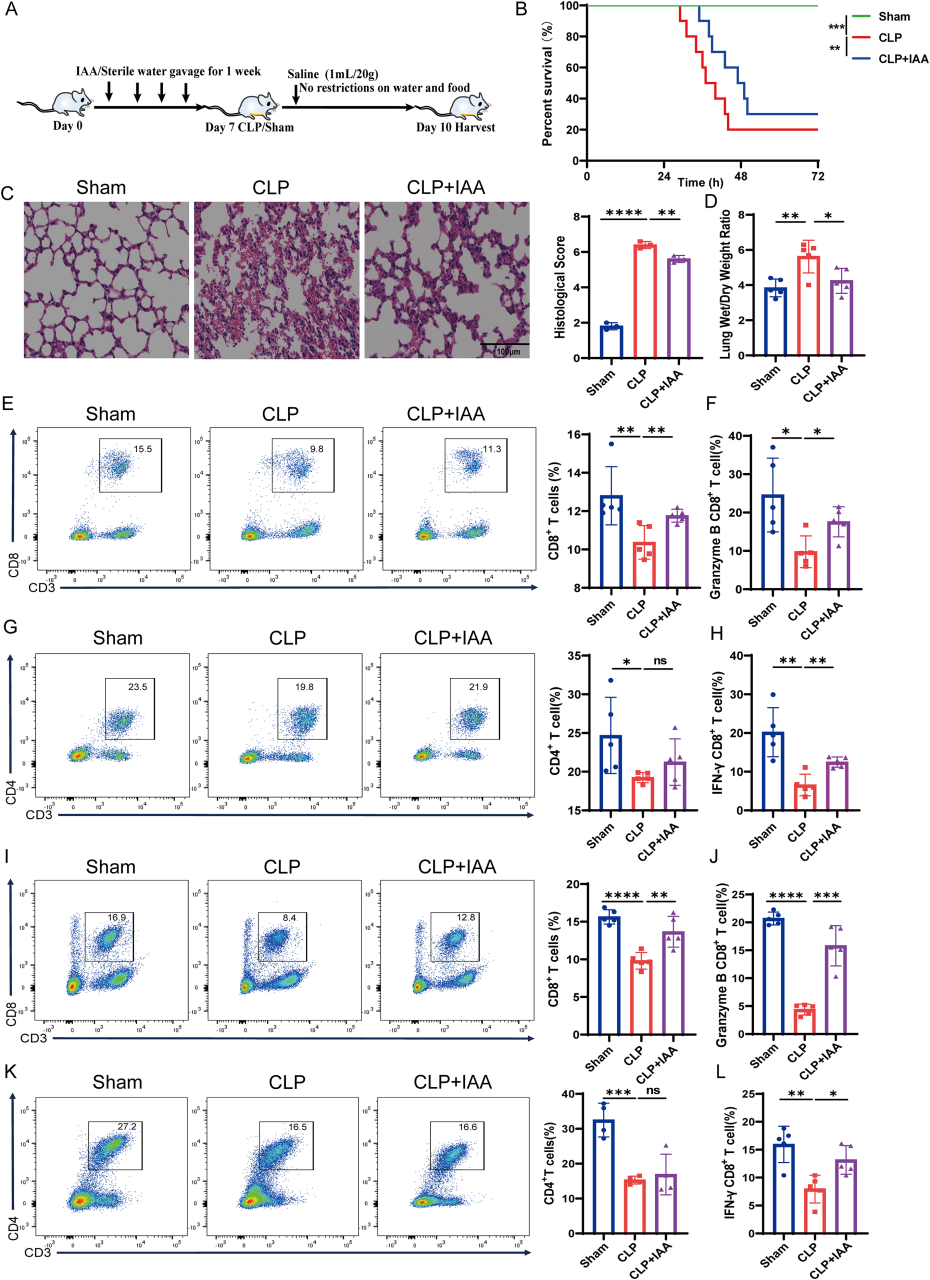
IAA Attenuates SI-ALI Through Enhanced CD8^+^ T Cell Effector Functions. **(A)** Experimental grouping and treatment flowchart for rats. **(B)** Kaplan-Meier survival analysis: Sham-operated, CLP-Induced SI-ALI, and IAA-Pretreated rats. **(C)** Representative H&E-stained rat lung sections and histological scores of Sham-operated, CLP, and IAA-Pretreated cohorts. **(D)** Comparison of the Lung Wet/Dry Weight Ratio of rats in each group. **(E–H)** Peripheral blood T cell frequency and cytokine production: CD3^+^CD8^+^ populations, CD3^+^CD4^+^ populations, IFN-γ^+^CD8^+^ subsets, and Granzyme B^+^CD8^+^ subsets in Sham-operated, CLP group, and IAA-Pretreated CLP Groups. **(I-L)** Splenic T cell frequency and effector function:CD3^+^CD8^+^ populations, CD3^+^CD4^+^ populations, IFN-γ^+^CD8^+^ subsets, and Granzyme B^+^CD8^+^ subsets in Sham-operated, CLP group, and IAA-Pretreated CLP Groups. * P<0.05; ** P<0.01; *** p<0.001; **** p<0.0001.

To determine the pathophysiological role of IAA in SI-ALI, we conducted an exogenous IAA supplementation intervention experiment. Administration of IAA 7 days before CLP significantly prolonged the overall survival of rats with CLP-induced SI-ALI, whereas no significant difference was observed in control rats (**Figures 5A, B**). Furthermore, histopathological analysis and lung wet/dry weight ratio revealed that ALI was significantly attenuated in rats receiving IAA supplementation compared with the untreated CLP group (**Figures 5C, D**). Flow cytometric analysis of blood and spleen samples revealed that both CD8^+^ T cell and CD4^+^ T cell populations were significantly reduced in the CLP group compared with the sham group. IAA pretreatment restored CD8^+^ T cell infiltration to near-normal levels, whereas no statistically significant change was observed in the proportion of CD4^+^ T cells compared to the CLP group (**Figures 5E, G, I, K**). These results suggest that IAA ameliorates SI-ALI-induced immunosuppression primarily by regulating CD8^+^ T cells. Furthermore, IAA pretreatment increased frequency of IFN-γ CD8^+^ T cells and granzyme B CD8^+^ T cells in SI-ALI rats (**Figures 5E–L**). Taken together, these results suggest that *Blautia*-derived IAA ameliorates SI-ALI by enhancing the number and function of host CD8^+^ T cells.

### 3.6 *Blautia*-potentiated CD8^+^ T-cell function attenuates SI-ALI

**Figure 6:**
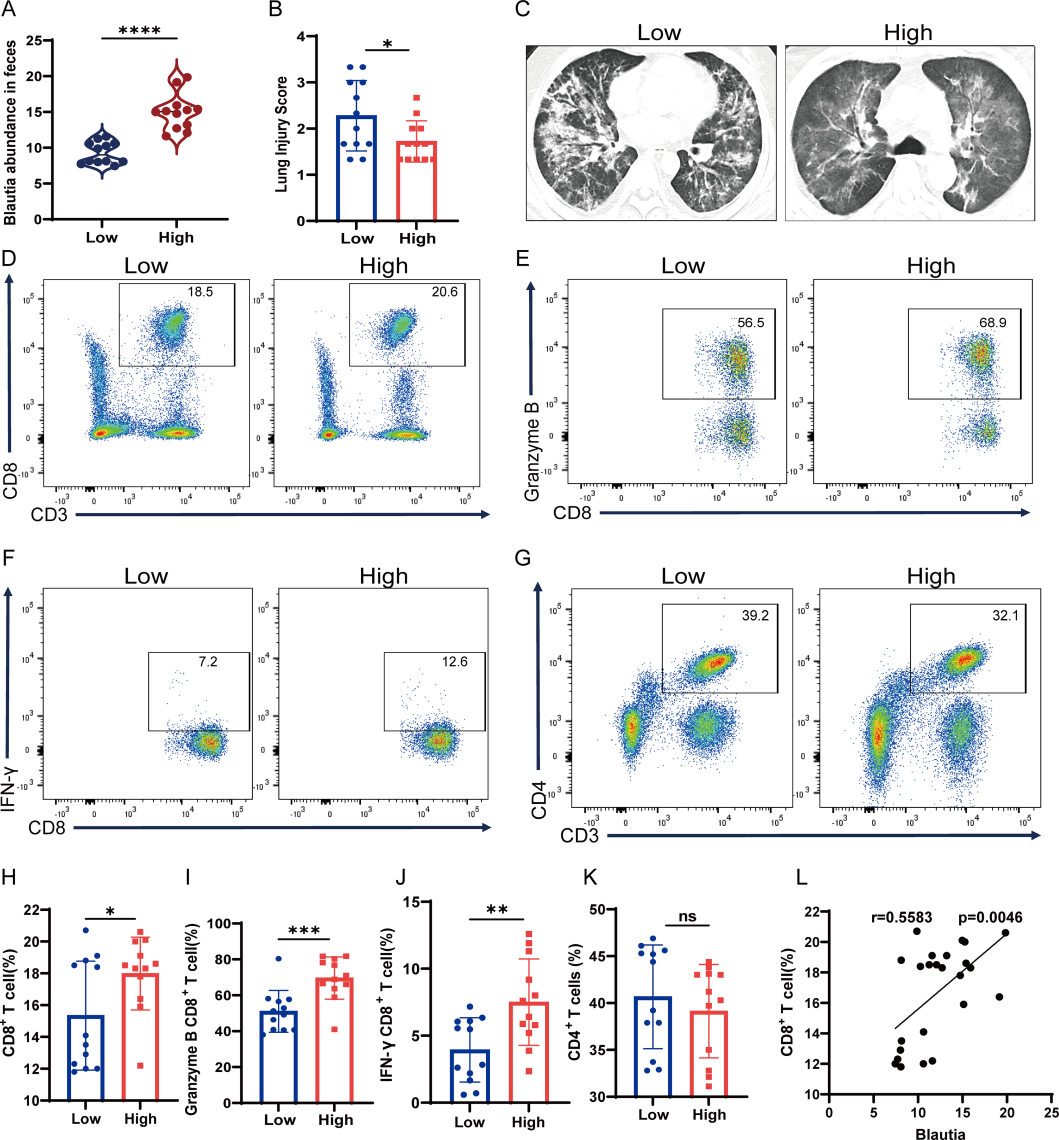
*Blautia*-Potentiated CD8^+^ T-Cell Recruitment Attenuates SI-ALI. **(A)** Abundance of *Blautia* in fecal specimens from SI-ALI patients. **(B, C)** Lung injury scores and chest CT manifestations of SI-ALI stratified by fecal *Blautia* levels. **(D-K)** Flow cytometric analysis of peripheral blood T lymphocytes in SI-ALI patients stratified by fecal *Blautia* abundance: CD3^+^CD8^+^ populations, CD3^+^CD4^+^ populations, IFN-γ^+^CD8^+^ subsets, and Granzyme B^+^CD8^+^ subsets. **(L)** Spearman correlation analysis between fecal *Blautia* levels and the count of peripheral blood CD8^+^ T cells. * P<0.05; ** P<0.01; *** p<0.001; **** p<0.0001.

To elucidate the mechanistic interactions between *Blautia* abundance, CD8^+^ T cell function, and lung injury severity in SI-ALI patients, we conducted a targeted clinical cohort study. Stool samples were collected from patients with sepsis and divided into groups with high and low *Blautia* abundance (**Figure 6A**). The high-abundance group had lower lung injury scores (**Figure 6B**). Chest computed tomography (CT) imaging confirmed this finding, showing less severe lung injury (**Figure 6C**). Flow cytometry analysis revealed a higher proportion of circulating CD8^+^ T cells in SI-ALI patients with high *Blautia* abundance compared with those with low *Blautia* abundance. Furthermore, SI-ALI patients with high *Blautia* abundance had higher expression of IFN-γ and granzyme B than those with low *Blautia* abundance (**Figures 6D–K**). *Blautia* abundance was positively correlated with peripheral blood CD8^+^ T cell levels (**Figure 6L**). Our results demonstrate that *Blautia* significantly enhances CD8^+^ T cell function, thereby alleviating SI-ALI-induced immunosuppression and reducing the severity of ALI in critically ill patients.

## 4 Discussion

The pathophysiology of SI-ALI features a concurrent state of excessive inflammation and immunosuppression (31). Dysregulated inflammation drives neutrophil extracellular trap (NET) release, complement cascade activation, and thrombotic inflammation. Conversely, immunosuppression manifests as accelerated immune cell apoptosis, T cell exhaustion, expansion of regulatory T cells and myeloid-derived suppressor cells (MDSCs), along with upregulated PD-1/PD-L1 checkpoint signaling (32, 33). Multiple studies demonstrate that intestinal and lung microbiota dysbiosis, driven by microbial communities and their metabolic byproducts, exacerbates ALI (34, 35). Immunometabolites serve as key mediators linking microbial ecology to systemic immune responses (36). Nevertheless, the specific contributions of gut microbiota and their metabolites to SI-ALI pathogenesis remain incompletely defined. We identified an association between *Blautia* abundance and SI-ALI progression in both clinical cohorts and a preclinical rat model. Critically, the *Blautia*-derived metabolite IAA increased the proportion of IFN-γ CD8^+^ T cells and granzyme B CD8^+^ T cells, thereby preventing lethal SI-ALI. Our work defines a novel “microbe-metabolite-lung immunity” axis underlying this protective mechanism.

Our results indicate reduced *Blautia* levels in SI-ALI patients compared to healthy controls. Subsequent analyses revealed significantly higher MDI scores in SI-ALI patients versus controls. This reduction may stem from non-sepsis factors, notably antibiotic-induced dysbiosis, or from SI-ALI pathophysiology itself. Specifically, SI-ALI drives excessive inflammation and microcirculatory dysfunction, causing intestinal epithelial damage and generating a redox-hostile microenvironment that suppresses *Blautia* growth (37).

Interestingly, our data revealed an increased relative abundance of several genera traditionally considered beneficial, such as Lactobacillus, alongside a reduction in Escherichia–Shigella in the CLP group. This observation does not imply an overall improvement of gut microbial homeostasis during sepsis. Rather, it likely reflects a transient and selective reshaping of the gut ecosystem in response to acute inflammatory stress. Given that 16S rRNA sequencing captures relative rather than absolute abundance, the apparent enrichment of certain taxa may result from the depletion of other dominant microbial populations. Moreover, some Lactobacillus species exhibit enhanced tolerance to inflammatory and hypoxic conditions, enabling their persistence during sepsis. Conversely, the reduced intestinal abundance of Escherichia–Shigella does not exclude its pathogenic role, as bacterial translocation rather than luminal expansion is a hallmark of septic progression.

*Blautia* represents a promising probiotic genus with therapeutic potential for diverse diseases. While mechanisms underlying its protective effects are increasingly elucidated, its specific roles in SI-ALI pathogenesis and immune regulation remain unclear. Crucially, multiple studies, including our own, demonstrate that *Blautia’s* viability is essential for its benefits, indicating that its bioactive metabolites critically maintain host homeostasis (38, 39). *Blautia* has been associated with immune surveillance and CD8^+^ T cell–related antitumor immunity (22). In contrast, its role in sepsis-associated immunopathology remains undefined. Here, in lethal SI-ALI induced by bacterial infection, we establish that *Blautia* promotes CD8^+^ T cell infiltration and amplifies production of effector molecules including IFN-γ and granzyme B, thereby improving survival and attenuating lung pathology in rats. The gut microbiota critically regulates host immunity and inflammatory responses, with CD8^+^ T cells serving as key mediators of inflammatory-immune crosstalk. Building on this foundation, we establish *Blautia* as a potent modulator of CD8^+^ T cell function that increased the proportion of CD8^+^ T cells while concurrently boosting production of effector molecules (IFN-γ and granzyme B). These findings expand our understanding of *Blautia*-driven immune regulation. Nevertheless, the precise mechanisms through which *Blautia* orchestrates CD8^+^ T cell functionality remain elusive and likely involve multifaceted pathways. In this model, the restorative effect appeared more pronounced for CD8^+^ T cells than for CD4^+^ T cells. This observation may reflect a model-specific pathophysiological emphasis on CD8^+^ T cell depletion and dysfunction during early, hyperinflammatory sepsis, or it may indicate that the primary immunomodulatory axis of Blautia-derived IAA acts more directly on the recruitment, survival, or effector differentiation of cytotoxic lymphocytes. The potential contribution of CD4^+^ T cell subsets, including T helper cell functions, to this protective mechanism warrants further investigation in future studies.

Accumulating evidence establishes intestinal microbiota-derived metabolites as critical mediators linking microbial ecology to systemic immune responses and immunotherapy efficacy (40, 41). Although gut microbiota have been linked to sepsis immunotherapy outcomes through modulation of disease progression and immune function, the therapeutic mechanisms of *Blautia*-derived metabolites in SI-ALI remained unexplored prior to this study. IAA has been reported to reduce oxidative stress and inflammation and promote intestinal barrier function. Furthermore, IAA exhibits potential therapeutic benefits in lung disorders, including ALI, pulmonary fibrosis, and chronic obstructive pulmonary disease (42–44). While several studies have reported protective or regulatory effects of microbiota-associated IAA in maintaining immune homeostasis, other reports—particularly in the context of pneumonia—have demonstrated deleterious effects associated with elevated or exogenously administered IAA (45). These seemingly discrepant findings suggest that the biological impact of IAA is highly context-dependent, influenced by disease setting, source and concentration of IAA, and the host immune state. Further mechanistic studies are therefore required to delineate the conditions under which IAA exerts beneficial versus harmful effects during infection and sepsis. In sepsis animal models, IAA administration significantly attenuated CLP-induced ALI, recapitulating the effects of *Blautia*. Collectively, these findings demonstrate that IAA represents a novel and promising metabolite-based therapeutic candidate for SI-ALI, whose mechanism involves the restoration of CD8^+^ T cell function.

While this study reveals novel therapeutic potential of *Blautia* for SI-ALI immunomodulation, clinical translation faces key limitations. First, preclinical models inadequately recapitulate human sepsis heterogeneity in pathology, underlying comorbidities, and infection sources. Second, mechanistic uncertainties persist regarding metabolite cross-regulation such as potential interactions between IAA and propionate, along with *Blautia’s* impact on other immune populations including neutrophil recruitment and natural killer (NK) cell function, requiring systematic investigation. Third, the initial human gut microbiome analysis was conducted in a small pilot cohort (n=5 per group), which precludes broad generalization and definitive statistical conclusions. Its primary purpose was to identify candidate microbial signatures, specifically the marked depletion of Blautia, in SI-ALI patients. This exploratory finding served as the crucial hypothesis-generating foundation that justified and directed our subsequent, more in-depth mechanistic investigations in a controlled animal model. The robust validation of Blautia depletion and the therapeutic efficacy of its supplementation/metabolite IAA in the preclinical model strengthen the biological plausibility of the initial human observation. A lack of exploration into the impact of Blautia/IAA on the long-term survival of SI-ALI patients, unverified functional differences between different Blautia strains, and the unclear specific receptors/pathways by which IAA regulates CD8^+^ T cells. In our subsequent studies, we will conduct multi-center, large-sample clinical trials to verify the association between Blautia and SI-ALI; and through *in vitro* cell experiments and *in vivo* animal experiments, we will verify the specific receptors and signaling pathways by which IAA regulates CD8^+^ T cells. Based on established literature regarding immunomodulatory microbial metabolites, the Aryl Hydrocarbon Receptor (AhR) represents a plausible candidate sensor for IAA and other tryptophan derivatives (46). Future studies employing AhR antagonists or AhR-deficient models are warranted to definitively establish its role in the observed IAA-mediated CD8^+^ T cell restoration.

A key limitation in interpreting the clinical microbiota data is the substantial impact of confounders, particularly broad-spectrum antibiotic exposure, which is known to drastically reduce obligate anaerobes like Blautia (37, 47). Other factors including parenteral nutrition and vasopressor support may further contribute to dysbiosis. While these treatments are intrinsic to sepsis management and difficult to disentangle from the disease’s effects, our experimental model, which controlled for such confounders, confirms that sepsis pathophysiology itself drives Blautia depletion. Furthermore, within our patient cohort uniformly exposed to these clinical confounders, the correlation between higher Blautia abundance and milder lung injury suggests its potential role as a resilient or protective taxon. Future studies with longitudinal sampling prior to antibiotic initiation are needed to definitively establish the causal trajectory.

In conclusion, our study establishes novel evidence for host-microbiome interactions during SI-ALI progression. By discovering IAAs associated with the genus *Blautia*, we expand the pathophysiological role of this genus. These findings position *Blautia* and its associated metabolite IAA as promising therapeutic strategies against lethal SI-ALI.

Nevertheless, the precise cellular and molecular mechanisms through which Blautia-derived IAA orchestrates CD8^+^ T cell recruitment and functional recovery remain to be fully elucidated and likely involve multifaceted pathways. Based on existing literature regarding microbial metabolite immunomodulation, several non-mutually exclusive mechanisms can be proposed for future investigation. First, IAA or its host-transformed derivatives may act directly on CD8^+^ T cells, potentially via the aryl hydrocarbon receptor (AhR) – a known sensor for various tryptophan metabolites like kynurenine and indole-3-aldehyde – to enhance cell survival, proliferation, and effector molecule transcription (46). Second, given the critical role of intestinal barrier integrity in sepsis-induced immunosuppression, the known beneficial effects of IAA on epithelial junction proteins (42) could reduce systemic bacterial translocation and the resultant immunosuppressive DAMPs/PAMPs, thereby indirectly relieving the brakes on T cell function. Distinguishing between these direct immunomodulatory and indirect barrier-protective effects will be a crucial next step in translating this ‘microbe-metabolite-immunity’ axis into targeted therapeutic strategies.

## Data Availability

The data presented in the study are deposited in the NCBI’s SRA (The Sequence Read Archive) database repository (https://dataview.ncbi.nlm.nih.gov/object/PRJNA1433168?reviewer=e3f7hkrponkg7udve96fcs0i34), accession number PRJNA1433168. The raw metabolic data has been stored in the MetaboLights database (https://www.ebi.ac.uk/metabolights/editor/MTBLS13984/files?reviewCode=63ac32bb-6fca-4217-a35c-454ad2440bb8), accession number MTBLS13984.
