# Supplementary material for: Indole-3-acetic acid derived from Blautia protects against sepsis-induced acute lung injury
Source: Front Immunol. 2026 Mar 23;17:1707493. doi: 10.3389/fimmu.2026.1707493 (PMC13050737; doi:10.3389/fimmu.2026.1707493)
Supplement: Supplementary file 1 [file Table1.docx]

**Supplemental Table 1. Demographic Data in SI-ALI and Controls Patients**

| **Variable** | **Controls (n=5)** | **SI-ALI (n=5)** | **P value** |
| --- | --- | --- | --- |
| Sex (male) | 4 (80%) | 2 (40%) | 0.524 |
| age | 62.0±6.6 | 55.2±5.8 | 0.462 |
| BMI | 21.2±2.1 | 23.2±2.0 | 0.507 |
| Diabetes（No） | 3(60%) | 1 (20%) | 0.524 |
| Chronic lung disease | 4 (80%) | 2 (40%) | 0.524 |
| Pneumonia | 3(60%) | 2 (40%) | 0.999 |
| Abdominal infection | 4(80%) | 1 (20%) | 0.999 |
| SOFA score (median [IQR]) | 0.0 [0.0, 0.0] | 4 .0[3.0, 5.0] | ＜0.001 |
| APACHE-II score (median [IQR]) | 4.0 [3.0, 5.0] | 14 .0[12.0, 18.0] | ＜0.001 |

The binary variables are described as counts and percentages and were evaluated by the Chi-squared test or Fisher’s exact test. Continuous variables of each group are presented as the mean ± SEM / Median [interquartile range]. Student’s t-test was used to compare the normally distributed continuous variables; Mann–Whitney U test is used to compare non-normally distributed continuous variables.
